# Comparative efficacy of three postpartum oral calcium formulations on metabolic dynamics, behavior, and performance in Holstein cows

**DOI:** 10.3389/fvets.2026.2024635

**Published:** 2026-10-30

**Authors:** Pelin Fatoş Polat Dinçer, Cenk Er, Ahmet İçigen, Sercan Hüseyin Bayendur, Abuzer Acar, Ömer Faruk Gökçecik, Ufuk Kaya, Egemen Erdem Öztürk, Mehmet Akköse, Tunahan Çomak, Mustafa Karaduman, Ahmet Ülker

**Affiliations:** 1 Department of Internal Medicine, Faculty of Veterinary Medicine, Eylül University, İzmir, Türkiye; 2 Department of Internal Medicine, Faculty of Veterinary Medicine, Kocatepe University, Afyon, Türkiye; 3 Parasitology Laboratory, Bornova Veterinary Control Institute, İzmir, Türkiye; 4 Department of Animal Science and Animal Nutrition, Faculty of Veterinary Medicine, Mustafa Kemal University, İzmir, Türkiye; 5 Department of Biochemistry, Faculty of Veterinary Medicine, Harran University, Şanlıurfa, Türkiye; 6 Department of Obstetrics and Gynecology, Faculty of Veterinary Medicine, Eylül University, İzmir, Türkiye; 7 Ülker Veterinary Clinic, İzmir, Türkiye; 8 Adil Aka Agriculture and Livestock, İzmir, Türkiye

**Keywords:** active vitamin D3, feeding behavior, milk yield, oral calcium bolus, parity, subclinical hypocalcemia

## Abstract

This study aimed to evaluate the comparative efficacy of three oral calcium supplementation strategies of distinct chemical formulations (an active vitamin D_3_-based bolus, a calcium chloride/sulfate-based bolus, and a calcium formate/acetate/sulfate-based bolus) on acute blood gas dynamics, mineral kinetics, and metabolic profiles (at 0, 12, 24, and 48 h postpartum), as well as their long-term impacts on annual milk yield, electronic sensor-monitored behavioral patterns, and reproductive performance in primiparous and multiparous Holstein cows. Ninety cows (45 primiparous, 45 multiparous) were assigned to three equal treatment groups. Blood parameters were analyzed using Linear Mixed Models with 0-h baseline values incorporated as covariates. Continuous 1-year follow-up data for milk yield, eating/rumination durations, and fertility were evaluated. The active vitamin D_3_-based bolus sustained the highest and most stable ionized calcium (1.36 ± 0.03 vs. 1.39 ± 0.04 mmol/L) and total calcium (8.27 ± 0.23 vs. 8.20 ± 0.24 mg/dL) levels at 48 h postpartum in primiparous and multiparous cows, respectively. The calcium chloride/sulfate bolus induced an early peak in ionized calcium at 12 h (1.23 ± 0.03 mmol/L for primiparous; 1.25 ± 0.04 mmol/L for multiparous), followed by a steady decrease. Meanwhile, the calcium formate/acetate/sulfate bolus showed a slower release pattern, marked by a transient ionized calcium at 24 h. Despite these distinct acute physiological responses, no significant long-term differences were observed among treatment groups regarding annual milk yield, rumination time, inseminations per pregnancy, or pregnancy rates (*p* > 0.05). Daily eating time was significantly longer in primiparous cows than in multiparous cows (*p* = 0.012). In conclusion, while oral calcium formulations offer specific short-term metabolic advantages during early transition, these transient benefits may not necessarily translate into long-term performance improvements, suggesting that oral calcium bolus supplementation may be more beneficial for meeting acute mineral requirements rather than providing extended productivity enhancements.

## 1 Introduction

The transition period from late pregnancy to early lactation is highly critical for maintaining metabolic homeostasis and reorganizing organ systems in high-yielding dairy cows (1). The sudden onset of colostrogenesis and milk synthesis at parturition rapidly depletes the circulating calcium pool. This process increases the daily calcium requirement by approximately threefold (1, 2). Periparturient hypocalcemia develops when bone mobilization and intestinal absorption fail to meet this sudden demand (3, 4). Ration management can successfully reduce the incidence of clinical milk fever below 5% (2, 5). Subclinical hypocalcemia (SCH) lacks obvious symptoms but maintains a high prevalence, making it a persistent hidden threat to herd health and farm economics. Indeed, because calcium is a fundamental macromineral governing neuromuscular function, immune responses, and metabolic homeostasis, its asymptomatic depletion triggers widespread multi-systemic impairments. Consequently, a substantial proportion of multiparous cows develop this invisible deficit, initiating a cascade of pathophysiological disruptions that compromise vital organ functions and performance (6, 7).

.Subclinical hypocalcemia reduces dry matter intake by suppressing smooth muscle motility. It disrupts insulin secretion, which in turn increases lipid mobilization. The condition also weakens immune functions by decreasing neutrophil phagocytosis capacity (3). This leads to a systemic mineral and metabolic imbalance characterized by declining levels of not only calcium but also phosphorus, magnesium, albumin, total protein, glucose, and iron (7). Postpartum calcium dynamics fall into three biological categories of transient, persistent, and delayed (1, 8). Transient SCH develops within the first 24 h postpartum and normalizes by days 2 to 4. This pattern is a natural indicator of the mammary gland adapting to high milk production (8). Conversely, persistent or delayed calcium deficiencies extending to the fourth day trigger metritis, ketosis, displaced abomasum, and significant milk yield losses (1). Evidence also shows that SCH can continue into later stages of lactation in higher parity cows (9). Because calcium is indispensable for smooth muscle contractility and immune competence, subclinical deficiencies may negatively influence reproductive performance by predisposing cows to uterine disorders such as metritis and subsequently contributing to fertility losses. Moreover, this compromised neuromuscular tone can suppress reticulorumen and abomasal motility, potentially leading to noticeable reductions in feeding activity and daily rumination times as behavioral reflections of metabolic imbalance (1, 8, 9).

Prepartum dietary adjustments and antepartum vitamin D_3_ or 25-hydroxyvitamin D_3_ administration are commonly used to prevent the negative effects of SCH (4, 10). Parenteral and oral calcium supplements are then administered during the postpartum period (5, 11). Intravenous calcium administration can cause rebound hypocalcemia following a sudden hypercalcemic peak (5). Subcutaneous applications might similarly lower serum calcium in the subsequent hours (11). Oral calcium boluses offer a more reliable alternative by providing gradual and sustainable absorption to elevate calcium levels. Their anionic effect further supports tissue sensitivity (4, 5). Combining these oral boluses with active vitamin D metabolites or acidogenic diets helps maintain serum calcium, lowers the somatic cell count, and reduces the risk of retained placenta (4, 10). The overall success of oral calcium administration still heavily relies on correct timing, chemical composition, and the specific parity group (2).

The primary objective of this study is to evaluate the effects of three chemically distinct oral calcium formulations on calcium absorption kinetics and metabolic profiles in transition dairy cows during the early postpartum acute phase at 0, 12, 24, and 48 h. The tested formulations consist of a bolus containing active vitamin D_3_, a bolus combining calcium chloride and calcium sulfate, and a third bolus containing calcium formate, calcium acetate, and calcium sulfate. We aimed to determine the interaction of these physiological responses with parity by comparing primiparous and multiparous cows. The study also comparatively analyzes how these early physiological changes influence long-term parameters such as annual milk yield, reproductive performance, and daily behavioral patterns including feeding and rumination times. We hypothesized that oral calcium boluses with differing chemical solubility and systemic acidogenic properties would exhibit distinct absorption dynamics in the acute postpartum period, differentially mitigating subclinical hypocalcemia across parity groups, and that formulations providing calcium bioavailability would favorably support subsequent rumination behavior, milk production, and reproductive outcomes.

## 2 Materials and methods

### 2.1 Ethical approval

All experimental protocols and animal handling procedures were reviewed and approved by the Institutional Experimental Animals Local Ethics Committee (HADYEK) of the Izmir/Bornova Veterinary Control Institute (Approval Date: June 2, 2025; Decision No: 19570218).

### 2.2 Study herd and animal management

The study was conducted in a commercial dry-lot Holstein dairy herd in Izmir, Turkey, between 2025 and 2026. The herd comprised approximately 3,102 lactating cows with an average daily milk production of 31.08 kg/day in the first lactation and 33.86 kg/day in the second lactation. During the final 3 weeks of gestation, primiparous and multiparous cows were transferred to prepartum close-up pens and fed a total mixed ration.

Immediately postpartum, calves were separated from dams and transferred to individual housing units. Cows were milked twice daily in a colostrum milking parlor for the first 4 days postpartum before moving to the fresh cow group for the subsequent 10 days. Routine reproductive and metabolic health monitoring was performed daily by herd veterinarians. To ensure methodological homogeneity, cows receiving any parenteral or oral calcium therapy or systemic medications outside the experimental protocol during the first 48 h postpartum were excluded from the study.

### 2.3 Inclusion criteria and subclinical hypocalcemia diagnosis

Subclinical hypocalcemia was defined and diagnosed according to the criteria established by Espiritu et al. (9). Specifically, cows exhibiting serum total calcium (tCa) concentrations ≤ 8.2 mg/dL at 0 h postpartum in the absence of overt clinical signs of parturient paresis were classified as subclinically hypocalcemic and included in the trial. This diagnostic cutoff was chosen to enhance diagnostic specificity beyond the immediate transition period and aligns with the lower boundary of the physiological reference range for adult dairy cattle (9). Cows diagnosed with subclinical hypocalcemia received no additional medical intervention or calcium rescue therapy throughout the monitoring period following initial detection. This non-intervention approach was maintained to avoid confounding effects and ensure that subsequent metabolic dynamics, behavioral patterns, and reproductive outcomes directly reflected the study’s experimental bolus formulations.

### 2.4 Experimental design, treatment groups, and control rationale

A total of 90 Holstein dairy cows exhibiting SCH were enrolled in a randomized block design stratified by parity: Primiparous (Lactation 1, *n* = 45) and Multiparous (Lactation 2, *n* = 45). Within each lactation cohort, animals were randomly assigned to one of three oral calcium supplementation treatment groups (*n* = 15 per group per lactation):

1. *Group 1-Phytogenic Calcitriol Glycosides Group (Active D)*: Administered an oral bolus containing plant-derived standardized calcitriol glycosides and ursolic acid (Active D®, Phytobiotics, Germany).
2. *Group 2- Mixed Organic–Inorganic Calcium Salts Group (Calstorm)*: Administered an oral bolus providing 45 g of elemental calcium combined with Vitamin D_3_ (Calstorm®, BayFeed, Türkiye).
3. *Group 3-Inorganic Calcium Salts Group (Bovikalc)*: Administered an oral bolus delivering 43 g of total calcium, composed of 71% fast-solubilizing calcium chloride and 29% sustained-release calcium sulfate (Bovikalc®, Boehringer Ingelheim, Germany).

All oral calcium treatments were administered immediately following postpartum blood sampling (0 h) in strict accordance with the manufacturers’ guidelines.

An untreated negative control group was intentionally omitted from the experimental design due to ethical and animal welfare constraints regarding subclinically hypocalcemic cows in a high-yielding commercial herd, where withholding calcium prophylaxis is medically contraindicated. Therefore, an active-comparator design was implemented to evaluate the comparative pharmacological efficacy of three distinct oral calcium supplementation strategies.

### 2.5 Sample size and a priori power and statistical analysis

An *a priori* power analysis was conducted to determine the required sample size for a repeated-measures design evaluating three treatment groups across four sampling time points (0, 12, 24, and 48 h postpartum). The analysis was parameterized for a within-between interaction effect (Time x Group), assuming a conservative repeated-measures correlation of 0.50 and a non-sphericity correction of 1.0. To detect a moderate effect size (Cohen’s *f* = 0.20) with an alpha level of *α* = 0.05 and a statistical power (1 - ß) of 0.80, a minimum total sample size of 45 animals per lactation cohort (15 animals per treatment arm) was required. Thus, enrolling 90 cows in total across both lactations provided sufficient statistical power (> 80%) to detect meaningful temporal variations and interaction effects in blood mineral parameters among treatment groups.

Time-dependent repeated measurements of blood parameters were analyzed using Linear Mixed Models (LMM). To account for baseline physiological and metabolic disparities between primiparous and multiparous cows, statistical models were constructed separately for each lactation cohort. Each physiological parameter served as a dependent variable, while treatment group (Group 1, 2, 3), sampling time (12, 24, 48 h), and the Group x Time interaction were included as fixed effects. Baseline values measured at 0 h were incorporated as covariates to adjust for individual initial variations.

To model the autocorrelation among repeated measurements, various covariance structures (e.g., compound symmetry, first-order autoregressive, unstructured) were evaluated using Akaike’s Information Criterion (AIC) and Bayesian Information Criterion (BIC). The first-order autoregressive [AR(1)] covariance structure provided the best goodness-of-fit, yielding the lowest AIC and BIC values, and was selected for the final repeated measures models.

Model residuals were assessed for normality using the Shapiro–Wilk test, and non-normally distributed parameters were log_10_ transformed prior to modeling. Pairwise comparisons of Estimated Marginal Means (EMMeans) were performed using Bonferroni’s post-hoc adjustment. Categorical data, including postpartum disease incidence and culling rates, were evaluated using Chi-square or Fisher’s exact tests. Data are presented as EMMeans ± Standard Error (SE), with statistical significance set at *p* < 0.05.

### 2.6 Blood sampling and laboratory analyses

Venous blood samples were collected via jugular venipuncture at 0, 12, 24, and 48 h postpartum. Point-of-care blood gas and electrolyte analyses were conducted immediately after collection using heparinized syringes and a portable blood gas analyzer (Epoc® Blood Analysis System, Siemens Healthineers, Erlangen, Germany). Measured and calculated parameters included partial pressure of oxygen (pO_2_), hematocrit (HCT), total hemoglobin (tHb), oxygen saturation (sO_2_), sodium (Na^+^), potassium (K^+^), ionized calcium (iCa^2+^), glucose, hydrogen ion concentration (cH^+^), base buffer [BB(B)], and calculated osmolality (mOsm).

For total calcium (tCa) determination, blood samples were collected into plain tubes without anticoagulant at 0, 12, 24, and 48 h postpartum. Samples were centrifuged at 3,000 x g for 10 min, and serum was stored at −20 °C until analysis. Total serum Ca concentrations were measured using a clinical chemistry analyzer (Fuji Dri-Chem NX500, Fujifilm Corporation, Tokyo, Japan). Furthermore, all measured biochemical and blood gas parameters strictly fell within the manufacturers’ validated analytical reportable ranges, requiring no sample dilution or off-scale extrapolation.

### 2.7 Behavior, rumination, milk yield, and health data collection

Continuous behavioral monitoring (rumination time, feeding duration, and active movement duration) was recorded using neck-mounted electronic sensors (GEA CowScout Sensor System, GEA Farm Technologies, Bönen, Germany). Animals were habituated to the collars as part of standard herd management. Daily milk yield, reproductive parameters (number of inseminations), culling rates, and clinical postpartum disorders (e.g., metritis, pyometra, retained placenta, displaced abomasum, and lameness) were recorded throughout a 1-year follow-up period by herd veterinarians using standard clinical diagnostic criteria.

## 3 Results

Table 1 details the effects of different oral calcium bolus administrations on blood calcium dynamics, acid–base balance, and hematological parameters in both primiparous and multiparous cows.

**Table 1:** Effects of different oral calcium boluses on blood calcium, acid–base, and electrolyte parameters in primiparous and multiparous cows at 0 (baseline), 12, 24, and 48 h.

Parameters	Lactation	Group	Hour 0 (Base)	Hour 12	Hour 24	Hour 48	P_Group_	P_Time_	P_GroupxTime_
pO_2_ (mmHg)	Primiparous	Group 1	36.33 ± 1.67	38.88 ± 1.49^a,x^	40.05 ± 1.49^a,x^	36.47 ± 1.49^a,x^	0.161	0.171	0.817
		Group 2	34.25 ± 2.43	37.03 ± 1.49^a,x^	39.19 ± 1.49^a,x^	36.44 ± 1.49^a,x^			
		Group 3	32.50 ± 0.65	34.50 ± 1.49^a,x^	35.25 ± 1.49^a,x^	33.00 ± 1.49^a,x^			
	Multiparous	Group 1	37.00 ± 3.00	36.19 ± 1.72^a,x^	37.64 ± 1.72^a,x^	36.08 ± 1.72^a,x^	0.505	0.407	0.551
		Group 2	34.67 ± 1.86	35.27 ± 1.83^a,x^	35.40 ± 1.83^a,x^	31.77 ± 1.83^a,x^			
		Group 3	35.75 ± 1.65	38.72 ± 1.64^a,x^	35.02 ± 1.64^a,x^	37.62 ± 1.64^a,x^			
Hct (%)	Primiparous	Group 1	29.33 ± 1.45	30.22 ± 0.76^a,x^	30.55 ± 0.76^ab,x^	29.30 ± 0.76^a,x^	0.203	0.187	0.528
		Group 2	29.00 ± 0.91	30.31 ± 0.76^a,x^	29.56 ± 0.76^ab,x^	28.06 ± 0.76^a,x^			
		Group 3	29.00 ± 1.08	29.23 ± 0.76^a,x^	27.65 ± 0.76^a,x^	26.98 ± 0.76^a,x^			
	Multiparous	Group 1	29.00 ± 1.53	26.74 ± 0.88^a,x^	26.85 ± 0.88^a,x^	27.74 ± 0.88^a,x^	0.122	0.510	0.854
		Group 2	29.00 ± 0.58	29.24 ± 0.93^a,x^	29.37 ± 0.93^a,x^	29.49 ± 0.93^a,x^			
		Group 3	27.75 ± 1.03	29.22 ± 0.85^a,x^	28.92 ± 0.85^a,x^	28.92 ± 0.85^a,x^			
tHb (g/dL)	Primiparous	Group 1	9.93 ± 0.44	10.30 ± 0.25^a,x^	10.38 ± 0.25^ab,x^	9.88 ± 0.25^a,x^	0.086	0.141	0.531
		Group 2	9.88 ± 0.32	10.36 ± 0.25^a,x^	9.94 ± 0.25^ab,x^	9.64 ± 0.25^a,x^			
		Group 3	9.83 ± 0.43	9.93 ± 0.25^a,xy^	9.38 ± 0.25^a,xy^	9.13 ± 0.25^a,x^			
	Multiparous	Group 1	9.87 ± 0.52	9.10 ± 0.28^a,x^	8.80 ± 0.28^a,x^	9.40 ± 0.28^a,x^	0.076	0.438	0.908
		Group 2	9.77 ± 0.15	9.92 ± 0.30^a,x^	9.94 ± 0.30^b,x^	10.00 ± 0.30^a,x^			
		Group 3	9.42 ± 0.38	9.93 ± 0.27^a,x^	9.86 ± 0.27^b,x^	9.85 ± 0.27^a,x^			
sO_2_ (%)	Primiparous	Group 1	67.33 ± 4.98	74.73 ± 2.42^a,x^	77.31 ± 2.42^ab,x^	73.31 ± 2.42^ab,x^	0.046	0.354	0.812
		Group 2	68.25 ± 3.90	73.34 ± 2.40^a,x^	73.84 ± 2.40^ab,x^	71.51 ± 2.40^ab,x^			
		Group 3	64.50 ± 1.55	67.84 ± 2.43^a,x^	69.01 ± 2.43^a,x^	64.01 ± 2.43^a,x^			
	Multiparous	Group 1	68.33 ± 4.10	71.15 ± 2.78^a,x^	75.81 ± 2.78^a,x^	73.48 ± 2.78^b,x^	0.479	0.443	0.509
		Group 2	65.67 ± 6.64	67.76 ± 2.94^a,xy^	70.01 ± 2.94^a,xy^	61.26 ± 2.94^a,x^			
		Group 3	70.50 ± 2.33	73.66 ± 2.63^a,x^	67.76 ± 2.63^a,x^	72.76 ± 2.63^b,x^			
Na (mmol/L)	Primiparous	Group 1	143.67 ± 0.88	140.49 ± 0.81^a,x^	139.41 ± 0.81^a,x^	139.24 ± 0.81^a,x^	0.177	0.141	0.655
		Group 2	144.50 ± 2.50	141.90 ± 0.84^ab,y^	138.40 ± 0.84^a,x^	139.15 ± 0.84^a,x^			
		Group 3	142.00 ± 0.91	143.53 ± 0.83^ab,y^	140.78 ± 0.83^a,x^	141.11 ± 0.83^a,x^			
	Multiparous	Group 1	139.67 ± 0.88	140.51 ± 0.93^a,x^	140.07 ± 0.93^a,x^	138.85 ± 0.93^a,x^	0.052	0.223	0.440
		Group 2	141.33 ± 1.45	143.90 ± 1.04^ab,x^	143.15 ± 1.04^a,x^	142.28 ± 1.04^ab,x^			
		Group 3	142.25 ± 2.06	141.31 ± 0.89^ab,x^	140.61 ± 0.89^a,x^	139.71 ± 0.89^ab,x^			
K (mmol/L)	Primiparous	Group 1	3.90 ± 0.06	4.05 ± 0.07^b,x^	4.06 ± 0.07^ab,x^	3.85 ± 0.07^a,x^	<0.001	0.039	<0.001
		Group 2	3.75 ± 0.12	3.45 ± 0.07^a,x^	3.63 ± 0.07^a,xy^	3.82 ± 0.07^a,xy^			
		Group 3	3.90 ± 0.04	3.92 ± 0.07^b,x^	3.86 ± 0.07^ab,x^	4.06 ± 0.07^a,x^			
	Multiparous	Group 1	3.70 ± 0.12	4.04 ± 0.08^a,x^	4.18 ± 0.08^ab,x^	3.98 ± 0.08^a,x^	0.260	0.448	0.134
		Group 2	3.87 ± 0.12	4.03 ± 0.09^a,xy^	3.86 ± 0.09^a,x^	4.24 ± 0.09^a,xy^			
		Group 3	3.88 ± 0.06	4.10 ± 0.08^a,x^	4.12 ± 0.08^ab,x^	4.15 ± 0.08^a,x^			
iCa (mmol/L)	Primiparous	Group 1	1.14 ± 0.03	1.22 ± 0.03^a,x^	1.26 ± 0.03^c,x^	1.36 ± 0.03^b,y^	0.033	<0.001	<0.001
		Group 2	1.14 ± 0.03	1.13 ± 0.04^a,y^	0.91 ± 0.04^a,x^	1.00 ± 0.04^a,x^			
		Group 3	1.15 ± 0.04	1.23 ± 0.03^a,y^	1.10 ± 0.03^b,x^	1.11 ± 0.03^a,x^			
	Multiparous	Group 1	1.18 ± 0.04	1.18 ± 0.04^a,x^	1.25 ± 0.04^ab,x^	1.39 ± 0.04^b,y^	0.589	0.002	0.001
		Group 2	1.13 ± 0.04	1.16 ± 0.04^a,x^	1.10 ± 0.04^a,x^	1.15 ± 0.04^a,x^			
		Group 3	1.15 ± 0.04	1.25 ± 0.04^a,xy^	1.21 ± 0.04^ab,xy^	1.11 ± 0.04^a,x^			
Glucose (mmol/L)	Primiparous	Group 1	3.37 ± 0.18	3.68 ± 0.13^a,y^	3.28 ± 0.13^a,x^	2.96 ± 0.13^a,x^	0.285	<0.001	0.033
		Group 2	3.65 ± 0.19	3.78 ± 0.13^a,y^	3.80 ± 0.13^ab,y^	3.32 ± 0.13^a,x^			
		Group 3	3.10 ± 0.15	3.67 ± 0.13^a,y^	3.61 ± 0.13^ab,y^	3.02 ± 0.13^a,x^			
	Multiparous	Group 1	3.50 ± 0.10	3.12 ± 0.15^a,x^	3.04 ± 0.15^a,x^	2.89 ± 0.15^a,x^	0.298	0.103	0.537
		Group 2	3.37 ± 0.18	3.47 ± 0.16^a,x^	3.27 ± 0.16^a,x^	3.37 ± 0.16^a,x^			
		Group 3	3.42 ± 0.10	3.42 ± 0.14^a,xy^	3.32 ± 0.14^a,xy^	3.01 ± 0.14^a,x^			
H^+^ (nmol/L)	Primiparous	Group 1	37.93 ± 0.90	36.07 ± 0.92^ab,y^	33.15 ± 0.92^a,x^	32.30 ± 0.92^a,x^	0.014	<0.001	0.003
		Group 2	35.80 ± 0.92	34.34 ± 0.91^a,x^	35.18 ± 0.91^a,x^	35.42 ± 0.91^b,x^			
		Group 3	37.83 ± 1.01	38.74 ± 0.92^ab,x^	38.43 ± 0.92^b,x^	38.41 ± 0.92^b,x^			
	Multiparous	Group 1	35.33 ± 0.88	34.60 ± 1.05^a,y^	31.64 ± 1.05^a,x^	30.38 ± 1.05^a,x^	0.061	0.046	0.126
		Group 2	35.33 ± 0.33	36.09 ± 1.11^a,x^	34.26 ± 1.11^ab,x^	36.29 ± 1.11^b,x^			
		Group 3	38.35 ± 0.74	36.22 ± 0.99^a,x^	37.17 ± 0.99^ab,x^	35.96 ± 0.99^b,x^			
BB(B) (mmol/L)	Primiparous	Group 1	45.43 ± 1.70	49.52 ± 0.87^ab,x^	51.06 ± 0.87^b,xy^	53.04 ± 0.87^b,xy^	0.013	0.018	0.005
		Group 2	48.62 ± 0.50	51.34 ± 0.86^ab,xy^	50.29 ± 0.86^b,xy^	48.88 ± 0.86^a,x^			
		Group 3	47.10 ± 1.76	47.17 ± 0.86^a,x^	47.09 ± 0.86^a,x^	48.45 ± 0.86^a,x^			
	Multiparous	Group 1	45.47 ± 2.05	50.17 ± 0.99^a,x^	53.85 ± 0.99^ab,y^	57.23 ± 0.99^b,z^	0.089	0.014	0.018
		Group 2	47.67 ± 1.25	50.01 ± 1.05^a,x^	50.56 ± 1.05^ab,x^	50.27 ± 1.05^a,x^			
		Group 3	48.47 ± 0.65	48.05 ± 0.94^a,x^	49.09 ± 0.94^a,x^	48.39 ± 0.94^a,x^			
mOsm (mOsm/L)	Primiparous	Group 1	288.07 ± 1.40	285.10 ± 1.54^a,x^	284.90 ± 1.54^b,x^	283.59 ± 1.54^a,x^	0.052	0.623	0.144
		Group 2	290.45 ± 3.51	285.56 ± 1.55^ab,y^	279.35 ± 1.55^a,x^	279.92 ± 1.55^a,x^			
		Group 3	287.65 ± 1.62	290.35 ± 1.55^ab,y^	284.83 ± 1.55^b,x^	285.10 ± 1.55^a,x^			
	Multiparous	Group 1	286.97 ± 1.58	283.60 ± 1.78^a,x^	282.93 ± 1.78^a,x^	280.93 ± 1.78^a,x^	0.155	0.528	0.536
		Group 2	284.60 ± 3.48	290.66 ± 1.94^ab,x^	288.49 ± 1.94^a,x^	287.84 ± 1.94^ab,x^			
		Group 3	287.90 ± 4.14	286.53 ± 1.71^ab,x^	285.14 ± 1.71^a,x^	283.25 ± 1.71^ab,x^			
Total Ca (mg/dL)	Primiparous	Group 1	7.55 ± 0.05	7.57 ± 0.23^a,x^	7.79 ± 0.23^ab,xy^	8.27 ± 0.23^c,xy^	0.106	0.127	<0.001
		Group 2	7.82 ± 0.16	6.76 ± 0.23^a,y^	6.74 ± 0.23^a,y^	6.07 ± 0.23^a,x^			
		Group 3	7.80 ± 0.35	6.73 ± 0.20^a,x^	7.58 ± 0.20^ab,xy^	7.24 ± 0.20^b,xy^			
	Multiparous	Group 1	7.26 ± 0.31	7.43 ± 0.24^a,x^	7.89 ± 0.24^ab,x^	8.20 ± 0.24^b,x^	0.940	0.104	0.016
		Group 2	7.55 ± 0.15	6.75 ± 0.25^a,xy^	7.05 ± 0.25^a,xy^	6.04 ± 0.25^a,x^			
		Group 3	7.68 ± 0.23	7.28 ± 0.22^a,x^	7.11 ± 0.22^ab,x^	6.68 ± 0.22^a,x^			

Data are presented as estimated marginal means ± standard error (EMMeans ± SE). Linear Mixed Model (LMM) analysis was conducted separately for primiparous and multiparous cows. The hour 0 (baseline) measurements were included in the model as covariates. P_Group_ represents the main effect of bolus administration. P_Time_ represents the main effect of time. P_Group × Time_ denotes the interaction between bolus administration and time. Values with different superscript letters (a, b, c) indicate significant differences between groups at the same time point. Values with different superscript letters (x, y, z) indicate significant time-dependent differences within the same treatment group.

### 3.1 Calcium parameters

A statistically significant Group × Time interaction emerged for Total Ca and iCa in both primiparous and multiparous cows (Primiparous Total Ca: *p* < 0.001, iCa: *p* < 0.001; Multiparous Total Ca: *p* = 0.016, iCa: *p* = 0.001). Total Ca levels in Group 1 primiparous cows began rising at 12 h and reached their peak by 48 h. Group 3 exhibited a distinct peak at 24 h before declining at 48 h. In contrast, Group 2 primiparous cows maintained relatively stable levels between 12 and 24 h but experienced a significant drop by 48 h (*p* < 0.05). For multiparous cows, Group 1 showed a steady upward trend. Their Total Ca levels at 48 h were significantly higher than those of Groups 2 and 3 at the same time point (*p* < 0.05). Groups 2 and 3 managed to maintain their Total Ca levels during the first 24 h, but both groups showed a marked decrease by 48 h (*p* < 0.05).

Ionized calcium dynamics revealed group-specific early peak times that differed from the Total Ca profiles. Consistent with the Total Ca pattern, iCa levels in Group 1 increased from 12 h onward and peaked at 48 h across both parity groups. Group 3 showed a rapid iCa peak at 12 h in both parity groups before declining in the subsequent hours. This occurred even though their Total Ca peak was delayed until 24 h. Following their 12-h values, iCa levels in Group 2 dropped sharply below baseline at 24 h, particularly in primiparous cows. These levels remained low for both parity groups through the end of the 48-h period (Table 1).

### 3.2 Acid–base balance

An analysis of acid–base parameters in multiparous cows showed a time-dependent increase in blood BB(B) levels for Group 1. These values remained relatively stable and close to baseline over the 48-h period in Groups 2 and 3. By 48 h, multiparous cows in Group 1 reached a BB(B) level of 57.23 ± 0.99 mmol/L, which was significantly higher than those recorded for Groups 2 and 3 at the same time point (*p* < 0.05). Hydrogen ion concentration in Group 1 decreased significantly from 12 to 48 h across both parity groups (*p* < 0.05). Group 1 primiparous cows exhibited a similar increasing profile for BB(B) (*p* < 0.05). Group 2 primiparous cows experienced a significant decline in BB(B) from 12 to 48 h (*p* < 0.05). Meanwhile, Group 3 displayed a fluctuating but generally horizontal trend for BB(B) values (Table 1).

### 3.3 Electrolytes, blood gasses, and metabolites

The Group × Time interaction for potassium concentrations was significant in primiparous cows (*p* < 0.001) but not in multiparous cows (*p* = 0.134). Blood potassium in Group 1 primiparous cows declined to 3.85 ± 0.07 mmol/L at 48 h. Conversely, potassium concentrations in Group 2 increased significantly between 12 and 48 h (*p* < 0.05).

No significant Group × Time interactions occurred among the calcium bolus treatments for sodium, osmolarity, pO_2_, sO_2_, hematocrit, or total hemoglobin values (*p* > 0.05). A significant Group × Time interaction was recorded for glucose levels in primiparous cows (*p* = 0.033). Glucose values in primiparous cows exhibited a gradual decline driven by the main effect of time (*p* < 0.001; Table 1).

### 3.4 Production parameters, behavior, and reproduction

The applied bolus groups did not significantly affect production parameters such as milk yield, rumination rate, feeding time, active movement, or the number of inseminations per pregnancy in either first or second lactation cows (*p* > 0.05). Different oral calcium boluses administered during the postpartum period exerted similar long-term effects on annual production performance, daily behavioral patterns, and reproductive parameters regardless of parity.

Comparing production, behavior, and reproductive parameters between the two parity groups revealed that lactation number significantly influenced average annual milk yield (*p* = 0.016) and daily feeding time (*p* = 0.012). Second lactation cows achieved notably higher annual milk yields compared to first lactation cows. In contrast, first lactation cows spent more time feeding daily than their multiparous counterparts. Parity groups showed no significant differences in daily rumination rate (*p* = 0.707), active mobility (*p* = 0.342), or number of inseminations per pregnancy (*p* = 0.404; Figure 1). Neither the bolus groups nor the lactation periods showed significant differences concerning the annual incidence of metabolic diseases (*p* = 0.405) or culling rates (*p* = 0.150).

**Figure 1:**
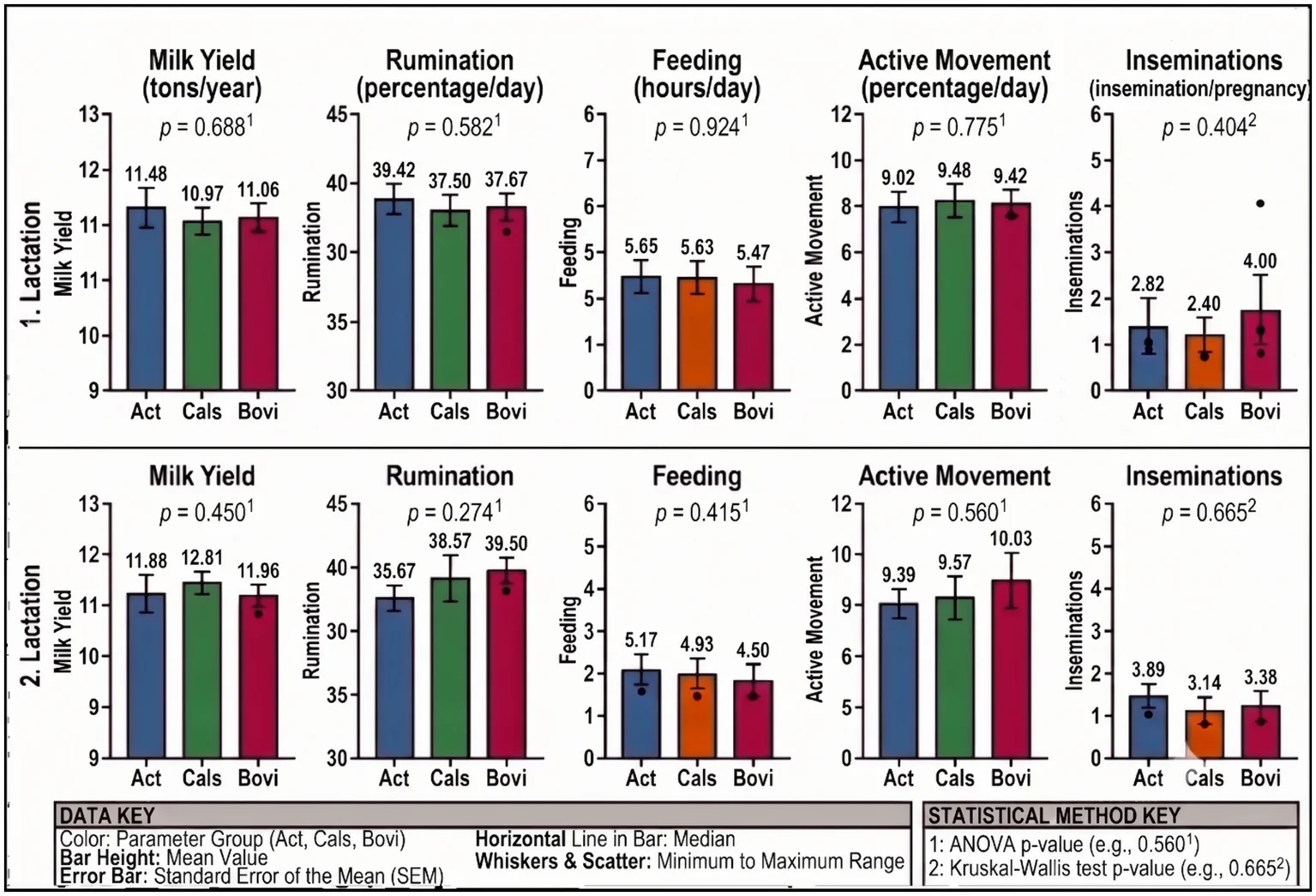
Comparison of annual production, behavioral, and reproductive parameters among supplement groups during the 1st and 2nd lactations.

## 4 Discussion

The postpartum transition period is a delicate phase in high-yielding dairy cows. The sudden increase in calcium demand for milk and colostrum synthesis severely challenges systemic homeostasis (12). When homeostatic mechanisms fail during this time, it paves the way for clinical and subclinical hypocalcemia along with interconnected metabolic and uterine diseases (13, 14). This study comparatively evaluated the effects of different postpartum oral calcium supplements on blood gasses, metabolic profiles, and long-term production parameters. The evaluated treatments included a bolus containing active vitamin D_3_, a bolus combining calcium chloride and calcium sulfate, and a bolus containing calcium formate, acetate, and sulfate.

Our findings indicate that the administered oral calcium formulations elicit different temporal responses regarding calcium absorption and plasma calcium kinetics. Both ionized and total calcium concentrations maintained a relatively high and stable course throughout the postpartum follow-up period in the active vitamin D_3_ bolus group. Active vitamin D metabolites reportedly stimulate active calcium transport across the intestinal mucosa and bypass renal hydroxylation constraints to support postpartum blood calcium stability in target tissues (15–17). This biological mechanism likely explains the sustainable and stable calcemic profile observed in the active vitamin D_3_ group.

Calcium chloride rapidly dissolves in the rumen to create a luminal calcium concentration gradient and is absorbed via passive diffusion (18, 19). The peak ionized calcium levels observed at 12 h in the calcium chloride and calcium sulfate group can be attributed to this rapid absorption mechanism. However, because this passive absorption across the rumen wall is short-lived and driven by a transient concentration gradient, the subsequent decline in calcium levels at 24 and 48 h likely reflects the rapid dissipation of this supplemental calcium pool alongside the continuous, heavy calcium drainage required for colostrogenesis and early lactation (20, 21). Previous studies have similarly emphasized that while calcium chloride-based boluses provide a temporary increase in blood calcium, they can also cause time-dependent fluctuations (18, 19).

Calcium levels generally remained lower in the bolus group containing calcium formate, acetate, and sulfate compared to the other groups. Ionized calcium hit its lowest point at 24 h, and total calcium showed a downward trend until 48 h in this group. Although calcium formate and acetate possess relatively high water solubility, their systemic bioavailability and absorption mechanisms across the gastrointestinal tract differ markedly from calcium chloride. Unlike calcium chloride, which rapidly elevates luminal ionized concentrations to force paracellular diffusion across the ruminal epithelium and induces a transient acidogenic response that enhances parathyroid hormone sensitivity, organic salts such as formate and acetate appear to provide lower bioavailability and lack the potent systemic acidifying drive required to provoke a rapid calcemic surge during the acute postpartum period (18).

Evaluating our findings based on parity revealed that the active vitamin D_3_ formulation tended to maintain relatively high and stable calcium levels in both first and second lactation cows. The active vitamin D component reportedly supports active calcium transport across the intestinal mucosa without being hindered by parity-related physiological constraints (15–17). This capability helps explain the stable calcemia observed in this group regardless of parity. Conversely, calcium concentrations in the calcium chloride/sulfate and calcium formate/acetate/sulfate groups were lower in first lactation cows compared to second lactation cows. Previous studies have established that primiparous cows display different calcium metabolism and physiological responses to oral supplements than multiparous cows (14, 20, 22, 23). Beyond these inherent physiological differences, our study found that first lactation cows spent significantly more time feeding daily than second lactation cows. Increased feeding duration and reduced intake per bite in primiparous cows often result from social stress and bunk competition, which can alter the absorption dynamics of both the ration and oral supplements. Our findings suggest that the metabolic response differences in primiparous cows may be linked not only to bone mobilization capacity but also to feeding behavior and stress-related physiological processes.

The effects of oral calcium salts on acid–base balance and blood gas parameters can vary depending on their electrolyte content and potential to alter the dietary cation-anion difference (22, 24). In our study, BB(B)- which serve as indicator of acid–base balance and buffering capacity-remained relatively low in the calcium chloride and calcium sulfate group. Correspondingly, hydrogen ion concentration was higher in this group. Acidogenic salts like calcium chloride and calcium sulfate are known to consume systemic buffering capacity during their absorption processes (12, 22). The parametric shift observed in this group likely originates from the acidogenic nature of the formulated salts. In contrast, the group receiving active vitamin D_3_ maintained their BB(B) levels over time while keeping the hydrogen ion load at a lower level. Oxygen saturation values also remained relatively higher in this group compared to the others. This suggests that calcium balance supported by active vitamin D_3_ might positively contribute to tissue perfusion and cellular respiration. The calcium formate, acetate, and sulfate group experienced a temporary increase in buffering capacity at 12 and 24 h, followed by a decline at 48 h. Analyzing the electrolyte kinetics revealed that potassium levels in primiparous cows decreased toward 48 h in the active vitamin D_3_ group, paralleling an alkalogenic effect. Meanwhile, potassium levels trended upward over time in the calcium formate/acetate/sulfate group. The gradual decline in postpartum glucose levels is a known physiological process associated with the gluconeogenic load of the transition period and energy metabolism adaptation at the onset of lactation (17, 21). Our findings regarding electrolyte kinetics and glucose likely reflect this underlying adaptation in energy metabolism.

Despite the physiological differences observed in blood gasses, acid–base buffering capacity, and serum calcium kinetics during the early postpartum period, the long-term effects of different oral calcium strategies on annual milk yield, rumination rate, feeding time, active movement, and inseminations per pregnancy did not reach statistical significance. This outcome aligns with data from extensive meta-analyses and field studies on the topic (13, 25–29). Prophylactic oral calcium bolus administrations reportedly have a limited impact on total lactation milk yield and pregnancy rates, particularly in herds with a low incidence of clinical hypocalcemia or those utilizing prepartum anionic dietary management (13, 29–31). Temporary metabolic and mineral improvements achieved early on often remain within subclinical limits, and homeostatic adaptation successfully occurs in cases that do not progress to clinical disease (25, 28, 32). The short-term metabolic advantages observed in our study likely failed to translate into long-term production and reproductive performance due to this exact homeostatic balancing process. Ultimately, these findings suggest that short-term changes in blood gas and mineral profiles during the early postpartum period may not be sole determinants of an animal’s long-term production potential and reproductive success.

## 5 Conclusion

In conclusion, among the different postpartum oral calcium formulations tested, the bolus containing active vitamin D3 tended to support a more sustained and stable calcium profile during the acute transition phase. The bolus based on calcium chloride and sulfate produced a rapid but transient increase in calcium, driven by the rapid solubility of calcium chloride. Conversely, the bolus containing calcium formate, acetate, and sulfate was associated with higher circulating glucose and specific hematological values. None of the evaluated calcium supplementation strategies conferred a distinct long-term advantage regarding annual milk yield, daily feeding or rumination durations, or reproductive performance parameters under the conditions of this study. Nevertheless, rather than indicating a lack of efficacy, these findings highlight that all three formulations successfully contributed to early postpartum mineral support through distinct physiological mechanisms, each offering specific clinical advantages for acute hypocalcemia management. These findings indicate that it may be more appropriate for veterinarians and herd managers to focus primarily on the immediate acid–base dynamics and acute mineral requirements of the early transition period when formulating postpartum oral supplementation protocols.

## Data Availability

The original contributions presented in the study are included in the article/supplementary material, further inquiries can be directed to the corresponding author/s.
